# Relation between the distance from the cementoenamel junction to the bone crest and the thickness of the facial bone in anterior maxillary teeth: A cross-sectional tomographic study

**DOI:** 10.4317/medoral.22802

**Published:** 2019-05

**Authors:** Julio Rojo-Sanchis, David Peñarrocha-Oltra, Miguel Peñarrocha-Diago, Regino Zaragozí-Alonso, José Viña-Almunia

**Affiliations:** 1 DDs, Master in Oral Surgery and Implant Dentistry, Oral Surgery Unit, Department of Stomatology, Faculty of Medicine and Dentistry, University of Valencia, Spain; 2 PhD, DDs, Assistant Doctor Professor of Oral Surgery, Oral Surgery and Implantology Unit, Stomatology Department, Faculty of Medicine and Dentistry, University of Valencia, Spain; 3 Prof. MD, PhD, Chairman of Oral Surgery, Director of the Master’s Degree in Oral Surgery and Implantology Unit, Stomatology Department, Faculty of Medicine and Dentistry, University of Valencia, Spain; 4 PhD, DDs Associated Professor of Oral Surgery, Oral Surgery and Implantology Unit, Stomatology Department, Faculty of Medicine and Dentistry, University of Valencia, Spain

## Abstract

**Background:**

The purpose of this cross-sectional study was to evaluate radiologically, the relation between the distance from the cementoenamel junction (CEJ) to the facial bone crest (FBC), and the facial alveolar bone (FAB) width at maxillary anterior teeth. A further aim was to assess if the CEJ-FBC distance had an impact in the prevalence to find a FAB thickness greater than one mm.

**Material and Methods:**

CBCT images were retrospectively obtained from the database of the Oral Surgery Unit of the University of Valencia. The teeth were divided in 3 groups according to the CEJ-FBC distance: Shorter (≤3mm), Middle (>3 ≤4.5 mm) and Larger (>4.5 mm). FAB thickness was measured by two different examiners at 1, 2 and 3 mm apical to the FBC. Normality of means were evaluated by Kolmogorov-Smirnov test and an ANOVA-type linear model was performed.

**Results:**

82 patients were included in the study, with 156 central incisors, 149 lateral incisors and 152 canines analyzed. A significant greater FAB thickness in Shorter (≤3mm CEJ-FBC) than Middle and Larger group was observed in all distances measured apical to the FBC. There was a significant inverse relation between the distance CEJ-FBC and FAB thickness at all distances measured. The prevalence of a FAB thickness equal or greater than one mm was 35.9% of all teeth analyzed from Shorter, 17.4% of Middle and 8.9% of Larger group at 1 mm apical to the FBC.

**Conclusions:**

When the distance from the CEJ to the FBC is augmented, thinner FAB thickness has to be expected in all teeth of the anterior maxilla. The prevalence to find a FAB thicker than one mm decreases as the distance from the FBC to the CEJ increases.

** Key words:**Facial bone, alveolar bone, cone-beam computed tomography, CEJ, maxillary teeth.

## Introduction

In a very high percentage of cases, facial alveolar bone (FAB) thickness at the anterior maxillary teeth is less than one mm (1-4). At these thin phenotypes, the first buccal coronal millimeters are only composed of bundle bone, a tooth-dependent structure which will be reabsorbed following tooth extraction (5,6). In humans, it has been demonstrated that when the FAB thickness is <1 mm, a mean height loss of 7.5 mm is going to happen after tooth extraction; while in cases of ≥1 mm thickness, 1.1 mm of vertical bone loss will occur (7). So the FAB thickness has an important relevance in the morphologic changes of the postextaction alveolus (8). In fact, some authors (9) decide the time from extraction to implant placement (immediate or early) depending on FAB thickness. Until now, the minimal FAB thickness required to avoid vertical crest resorption has not been established (10).

The distance from the cementoenamel junction (CEJ) to the facial bone crest (FBC) might be a factor to take into account. Some authors (4,11) have observed a direct relationship between this distance and the age of the patient. Other important factor is the tooth to be analyzed; Wang *et al.* (12) reported that the CEJ-FBC distance was greater at maxillary canines than at incisors. It also has been shown that this distance increases with systemic diseases and smoking habit (13).

Some studies (4,11,14,15) have found that FAB thickness at maxillary anterior teeth is thinner at apical level than at the first coronal millimeters of the FBC, but other authors (1,16) have reported opposite results, i.e thinner FAB at the coronal crest. It is well known that periodontal disease is characterized by loss of connective tissue attachment and alveolar bone destruction that starts at coronal levels (17). To our knowledge, no studies have focused on the relation between FAB thickness and CEJ-FBC distance at maxillary anterior teeth.

The aim of the present study was to evaluate the relation between the distance from the CEJ to the FBC and the FAB thickness at maxillary anterior teeth. A further aim was to assess if the CEJ-FBC distance had an impact in the prevalence to find a FAB thickness greater than one mm. This article was written following the STROBE statement (18) for improving the quality of observational studies.

## Material and Methods

The study protocol was submitted to and approved by the Ethics Committee of the University of Valencia, Spain (procedure no. H145639215058). The CBCT images were retrospectively obtained from the database of the Unit of Oral Surgery in the University of Medicine and Odontology of Valencia. All CBCT scans had been performed for diagnostic or treatment plan purposes between September 2013 and March 2017. Images were obtained using the same machine and general adjustment settings. All of the scans were ordered in the course of routine dental care. The following inclusion and exclusion criteria were set.

-Inclusion and exclusion criteria

CBCTs from patients that met the following inclusion criteria were included: subjects aged 18 years or older, no history of orthodontic treatment either marked tooth resorption.

Patients younger than 18 years old, smokers of more than 20 cigarettes a day, patients with dental implants, endodontic or prosthetic restorations at anterior maxillary teeth were excluded. Patients having history of trauma or receiving osseous/regeneration procedures were excluded.

-Patient data collection

The following variables were collected for each patient using a predetermined study protocol: sex, age, smoking habit, CBCT purpose, distance CEJ-FBC, thickness of the FAB at central incisors (CI), lateral incisors (LI) and canines (C).

-Radiographic image analysis

**Figure 1:**
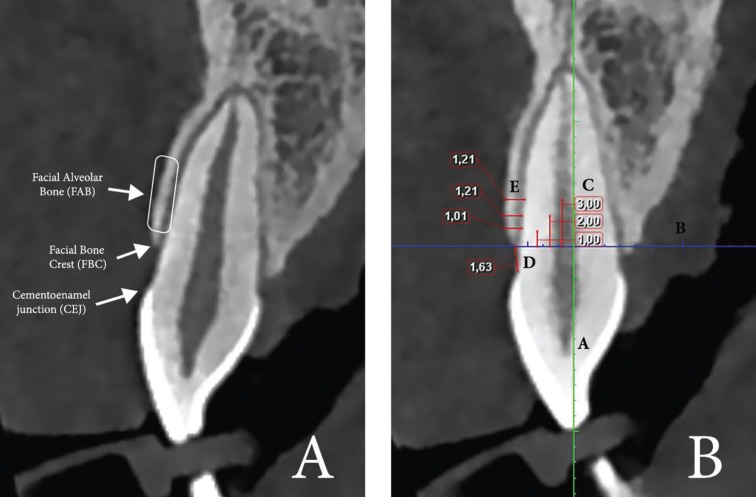
A. Location of anatomic regions (Facial alveolar bone, Cementoenamel junction and Facial Bone Crest) identified at the sagittal slice. B. Measurements at the sagittal slice. Line A: line (green) of reference along the longitudinal axis of the teeth. Line B: line (blue) of reference perpendicular to the long axis of the teeth at the level of the FBC. Lines C: lines of reference at 1, 2 and 3 mm below the FBC and parallel to Line A. Line D: measurement from the CEJ to the FBC. Lines E: measurements of the FAB thickness, parallels to line B, with reference to lines C. This image shows that the FAB thickness at 1, 2 and 3 mm apical to the FBC measures 1.01, 1.21 and 1.21 mm respectively.

The CBCT images were acquired using Planmeca ProMax 3D (Software 2.3.1. R TM Planmeca Romexis Helsinki, Finland) with a voxel size of 0.4 mm, 150 mSv, 90 kV, 10.0 mA, and a field of view (FOV) of 4 x 4 cm. The scans were uni or bilateral depending on their diagnostic purpose. All images were analyzed with the same computer and same monitor (Eizo Nanao Flexscan HD2442W with a resolution of 1280 x 1024 pixels). To detect the slice location to perform the measurements, we proceeded as follows: The alveolar crest was located at the axial plane and a buccopalatal slice was traced at the middle of the root. Then, the long axis of the root was determined at the coronal slice. Anatomic locations (FAB, CEJ, FBC) taken as reference points are illustrated at Figure 1a. The measurements from the CEJ to the FBC and of the thickness of the FAB were performed at the sagittal plane (Fig. 1b) as reported by Rojo-Sanchis *et al.* (19) For additional analysis, the subjects were divided into 3 groups according to the CEJ-FBC distance (Shorter group: ≤ 3 mm; Middle group: >3 and ≤ 4.5 mm; Larger group: > 4.5 mm) ( Table 1). Physiological bone levels range from 1 to 3 mm apical to the CEJ (20,21) (Shorter group); Middle and Larger groups correspond to teeth that had slightly or great loss of periodontal attachment. Three measurements were then made parallel to this perpendicular line 1, 2 and 3 mm apical to the FBC. The relation between the distance CEJ-FBC and the FAB thickness was analyzed in groups and in continuous variable. All teeth and images were measured by two different examiners (JR, RZ); discrepancies between the first two examiners were resolved consulting a third adviser (JV).

**Table 1:**
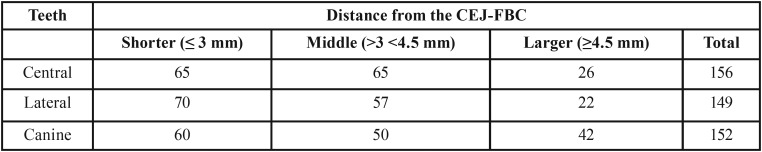
Sample description divided into groups depending on the distance CEJ-FBC.

-Examiner calibration

The mean difference between the measurements of both observers was 0.016 ± 0.045 mm (SE), without appreciating statistically significant bias through the paired t-test (*p*> 0.05). The Dahlberg statistic, took the value 0.26 mm and the average intra-class correlation coefficient was 0.80, so a high degree of inter-examiner reproducibility can be accepted.

-Statistical analysis

A previous pilot study in 37 patients was carried out to determine the sample size. A minimum of 80 patients were needed for an ANOVA F test to achieve a power of 90% in order to detect as significant an effect size as observed in the pilot (f = 0.35). The objectives were addressed through a parametric approach, 95% confidence intervals are provided for the estimation of mean dimensions (SPSS, v.24.0 for Windows, IBM, Chicago, IL).

The inter-subject correlation was controlled by inferential analysis. Simple linear regression was used to analyze the relationship between FAB thickness and CEJ-FBC distance, calculating prediction intervals and evaluating goodness of fit from the R2 determination coefficient. An ANOVA-type linear model compares the averages of thickness at three distance intervals. Assuming one mm as cut value to size the thickness, a logistic regression is applied to study the probability that this amount will be exceeded as a function of distance. Odds ratio estimates are obtained to quantify the impact of changes in distance on that probability. The level of significance used in the tests was 5% (α = 0.05).

## Results

-Sample description

A total of 82 CBCT images were used for this study, including images from 37 men and 45 women with a mean age of 39.6 years (age range 18-60 years). CBCT were performed for the following purposes: palatally impacted canines (6), impacted wisdom molars (24), apical surgery (13) or implant planning (39). Thus, a total of 156 CI, 149 LI and 152 C were analyzed ( Table 1). Nineteen patients were heavy smokers (> 10 cigarettes/day), 27 were light smokers (≤ 10 cigarettes/day) and 36 were nonsmokers.

-Radiographic outcomes

**Figure 2:**
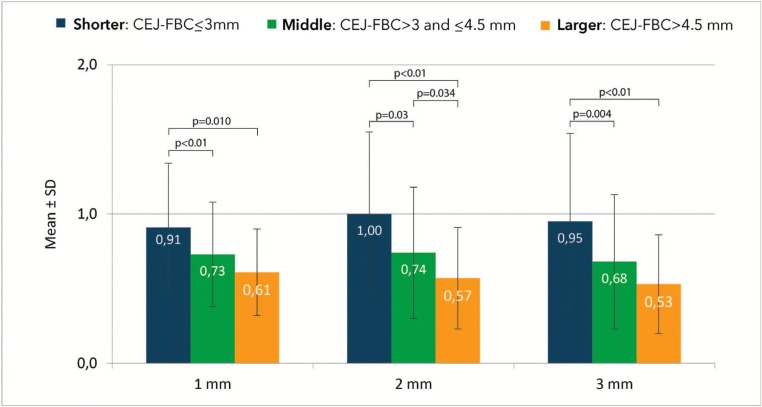
FAB thickness (mm) at 1, 2, 3 mm from the FBC divided by groups according to the CEJ-FBC distance: Shorter: ≤ 3 mm; Middle: 3-4.5 mm; Larger: ≥4.5 mm. Mean greater FAB thickness in Shorter than Middle (*p*>0.05) and Larger group (*p*<0.01) was observed in all distances measured apical to the FBC. Between Middle and Larger group only at 2 mm below the FBC the differences were significant (*p*=0.034), with mean greater FAB thickness in Middle group.

FAB thickness for different teeth and groups is presented in  Table 2. Mean greater FAB thickness in Shorter (≤3mm CEJ-FBC) than Middle (1 mm *p*=0.01; 2 mm *p*=0.003; 3 mm *p*=0.004) and Larger group (*p*<0.001) was observed in all distances measured apical to the FBC (Fig. 2). Between Middle and Larger, only at 2 mm below the FBC the differences were significant (*p*=0.034), with mean greater FAB thickness in Middle group.

**Table 2:**
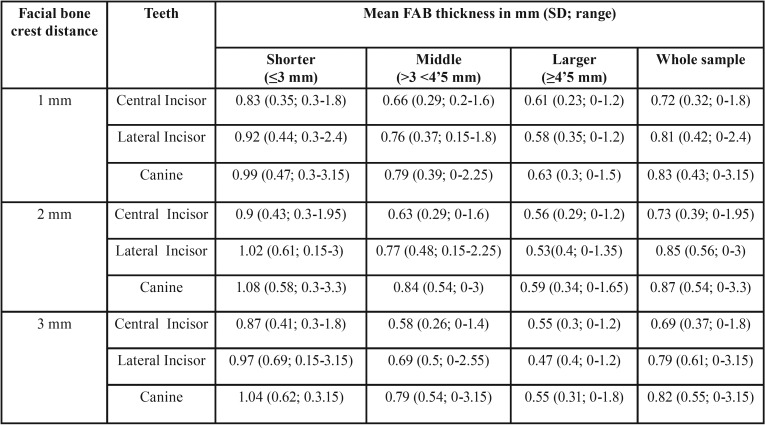
FAB thickness (mm) at 1, 2 and 3 mm from the FBC divided by teeth and groups.

**Figure 3:**
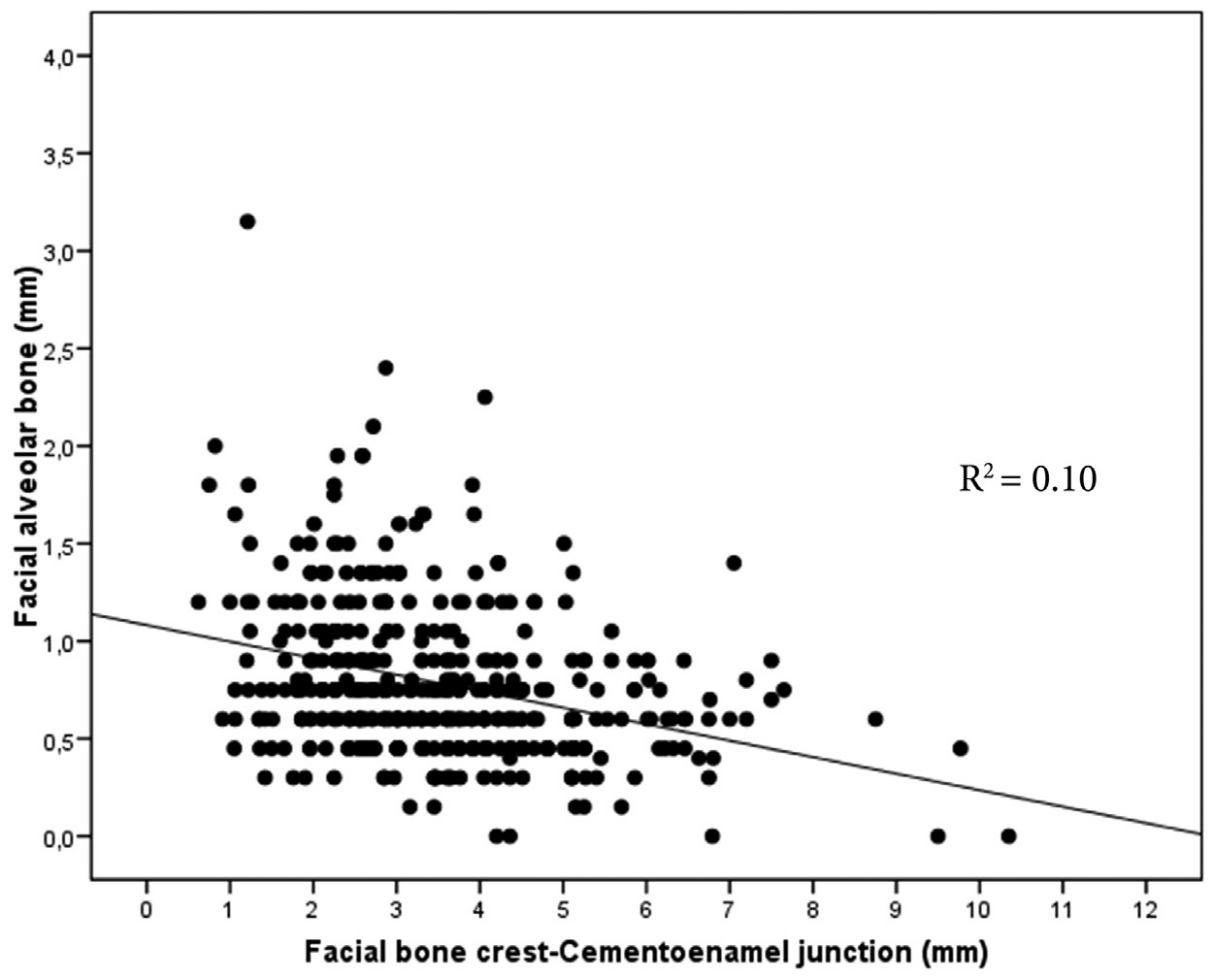
Linear regression analysis. A statistically significant (*p*<0.001) inverse relationship between CEJ-FBC distance and FAB thickness of weak intensity (R2=0.10) can be observed.

For any teeth and level of measurement from the FBC, there was a significant inverse relationship between FAB thickness and CEJ-FBC distance. A simple linear regression model shows that, this relationship is statistically significant (*p* <0.001). However, the relationship can only be considered of weak intensity (r=-0.318; R2=0.10) (Fig. 3).

The amount of teeth that presented FAB thickness equal or greater than one mm, also varied according to the CEJ-FBC distance. A FAB thickness ≥ 1 mm was observed in 35.9% of teeth from Shorter, 17.4% of Middle and 8.9% of Larger group, from all teeth analyzed (CI, LI and C) at 1 mm apical to the FBC. The proportion of teeth that reached this FAB thickness from Shorter group were significantly greater with respect to Middle (*p*=0.009) and Larger group (*p*<0.01). Between Middle and Larger group differences are at the limit to be significant (*p*=0.066). Thus, the prevalence to have a FAB thicker than one mm is higher when the distance from the CEJ to the FBC is smaller.

## Discussion

The main purpose of this study was to analyze the relation between the CEJ-FBC distance and the FAB thickness at maxillary incisors and canines. A secondary aim was to assess if the CEJ-FBC distance had an impact in the prevalence to find a FAB thickness greater than one mm. An inverse relationship between the CEJ-FBC distance and the FAB thickness was observed. According with the results obtained in the present study, a thinner FAB at the anterior maxillary teeth can be expected if the CEJ-FBC distance is higher; however it would be difficult to predict FAB thickness just taking into account this fact. Another finding of the present study was that the probability to find a FAB thickness equal or greater than one mm was higher as the CEJ-FBC distance decreases. To our knowledge no other study has related these two variables.

According to the anatomy of the FAB at anterior maxillary teeth, a recent study analyzed CBCT images of 3618 teeth including incisors, canines, premolars and first molars. The mean FAB thickness at anterior teeth was 0.9 mm and only 1.8% reached 2 mm in thickness (4). Januario *et al.* (2) reported that maxillary incisors and canines in most locations presented ≤ 1 mm, and that close to 50% had a thickness ≤ 0.5 mm. Braut *et al.* (1) obtained a thick FAB (≥ 1 mm) in approximately 10% of the teeth analyzed. The results of the present study are similar to those reported in the literature; showing a mean FAB thickness of 0.72 mm in CI, 0.81 mm in LI and 0.83 mm in C. Different teeth presented different FAB thickness. Authors (4,12) who have measured anterior and posterior areas have reported a significant increase in FAB thickness from incisors to premolars. In a previous study, we also observed that first premolars had significant greater FAB thickness than second premolars (19). Another factor to take into account when analyzing the anatomy of the alveolar process at maxillary anterior teeth, is the relation between the angulation of the root axis and the basal bone. López-Jarana *et al.* (22) reported mean values of 11.67 ± 6.37° for incisors and 16.88 ± 7.93° for canines, which means that anterior maxillary teeth angle in the alveolar process makes them to be in close contact with the FAB.

Buccal bone resorption after tooth extraction differs among studies, individuals and sites. Factors implicated in this variation includes the presence and absence of existing infection, flap versus flapless extraction, the extent of trauma during extraction, and the thickness of the FAB prior to the extraction (10,16). In a prospective CBCT study (7), 8 weeks after tooth extraction, a mean vertical bone loss of 7.5 mm of the FAB was observed in the presence of one mm or less FAB thickness. In contrast, only a mean vertical bone loss of 1.1 mm was observed in patients with a thick wall phenotype. Ferrus *et al.* (23) performed a clinical study of post-extraction implant placement filling the gap with biomaterial. They observed smaller vertical resorption in sites with thicker FAB, however at sites with a thin FAB (<1mm), there was a substantial vertical loss (1.2-2.1mm) (23). Thus, FAB thickness plays an important role in post-extraction alveolus dimensional changes and implant treatment plan.

Research has shown that significant bone modeling activities occur during the first 2 months of postextraction healing (6). Bone modeling in single extraction sites is mainly localized to the central aspect of the FAB, whereas proximal aspects are well maintained by the periodontal ligament of the adjacent teeth. For that reason, our study was focused on this central area. The dimensional bone and soft tissue alterations following tooth extraction in the anterior maxilla have a significant impact on the esthetic outcome of implant-supported restorations (24).

Local factors such as history of periodontal disease, gingival recession (25) or non-carious cervical lesions (26) increase CEJ-FBC distance, but also systemic factors such as age, smoking habits (27), depression, diabetes, asthma, hypertensive and thyroid disorders (13) have influence in the CEJ-FBC distance. Some studies have observed an increase of this distance at 50 years or older individuals (11,12). So there are local and systemic factors that may influence the CEJ-FBC distance, and according with the present study in anterior maxillary teeth, as this distance increase, the FAB thickness decreases.

It is important to note that the data in our study is based on CBCT scans from patients of a specific region. Socket dimension anatomy of subjects of different ethnicities may be very different from those reported herein.

The present study concludes that there is a significant inverse relationship between the distance from the FBC to the CEJ and the thickness of the FAB for different teeth in the anterior maxilla. As the FBC-CEJ distance increase, the thickness of the FAB decreases. The prevalence to find a FAB thicker than one mm decreases as the distance from the FBC to the CEJ increases.
